# Rare Idiopathic Adult Intussusception: A Case Report

**DOI:** 10.7759/cureus.52022

**Published:** 2024-01-10

**Authors:** Alexandra Frazier, Raymond Mbah, John T Williams

**Affiliations:** 1 Surgery, Trinity Medical Sciences University, Macon, USA; 2 General Surgery, Piedmont Macon Medical Center, Macon, USA

**Keywords:** abdominal pain, intussusception, small bowel obstruction, small bowel intussusception, adult intussusception, emergency exploratory laparotomy, idiopathic intussusception

## Abstract

Intussusception is highly uncommon in adults, with most cases caused by a pathological lead point that requires surgical resection. This case presentation highlights a rare example of idiopathic intussusception in a young male adult. Our patient is a 23-year-old African American male who presented at the Piedmont Macon Medical Center emergency department in February 2023 with acute-onset severe periumbilical pain, nausea, vomiting, and diarrhea. Computed tomography (CT) imaging was inconclusive, and his diagnosis of an ileo-ileal intussusception in the distal ileum was made during an exploratory (diagnostic) laparoscopy. Based on a visual inspection of the bowel demonstrating no evidence of inflammation, adhesions, lesions, ischemia, or a pathological lead point, manual reduction without resection was indicated. While intussusception is rare in adults, it is an important clinical prognosis that should be carefully considered in the differential diagnosis.

## Introduction

Intussusception is classically defined as the telescoping of a proximal segment of the bowel into a distal segment [1-6]. It is the most common cause of intestinal obstruction in children under three years of age [7,8]. It is rare in adults, accounting for only 1%-5% of bowel obstructions [4-7] with a mean age of 50 years old [9]. Compared to pediatric intussusception, adult intussusception is distinct in that 90% of cases are secondary to a pathological lead point, not limited to polyps, carcinomas, strictures, adhesions, and Meckel diverticulum [4-7]. Less commonly, adult intussusception is idiopathic, accounting for only about 10% of cases [6]. Most adult patients present as an emergency with a clinical presentation similar to intestinal obstruction [4]. However, the disparity in vague presenting symptoms makes diagnosis difficult and may only be established once in the operating room. Radiographic imaging such as a plain abdominal X-ray, abdominal CT scans, abdominal magnetic resonance imaging (MRI) scans, and ultrasound can be advantageous in diagnosing.

Considering that the vast majority of intussusception in adults occurs secondary to a benign or malignant neoplasm, surgical intervention is typically required for treatment [7]. Recent studies suggest a more selective approach to bowel resection that takes the location of the intussusception and the pathology of the underlying cause into consideration [1,10]. In this paper, we present a rare case of idiopathic intussusception in a young adult male treated intraoperatively via manual reduction.

## Case presentation

**Figure 1:**
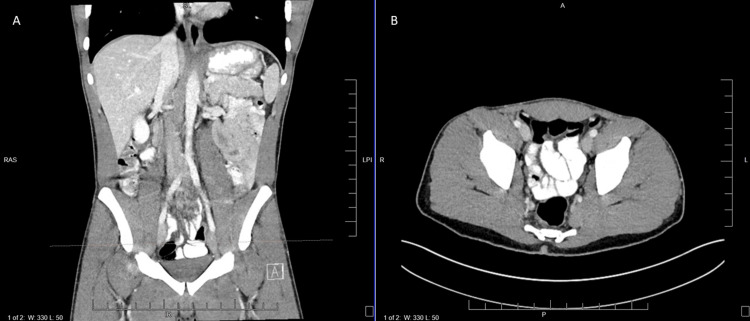
CT abdomen and pelvis with contrast from January 1, 2023 A) CT abdomen and pelvis coronal view. The dotted orange line indicates the corresponding level in Panel B. B) CT abdomen and pelvis axial view.

On February 22, 2023, a 23-year-old male presented to the emergency department with complaints of acute-onset persistent epigastric pain. He also complained of accompanying non-bloody diarrhea, nausea, and vomiting spanning a 20-hour duration from onset. Two months prior, on January 1, 2023, the patient visited the emergency room with similar complaints of abdominal pain. However, CT imaging (abdomen/pelvis with oral and IV contrast) at that time revealed no acute abnormality within the abdomen or pelvis (Figure 1).

Lipase and troponin assessments were within the reference range. Abnormal lab results from his complete blood count (CBC) and comprehensive metabolic panel (CMP) are shown in Table 1. Other than that solitary visit, the patient’s past medical history was unremarkable.

**Table 1:** Abnormal lab results from January 1, 2023

Component	Reference Range & Units	01/01/23
Neutrophils Relative	41.0% to 79.0%	80.0
Lymphocytes Relative	15.0% to 48.0%	9.8
Neutrophils Absolute	2.2 to 4.8 10*3/μL	7.7
Lymphocytes Absolute	1.3 to 2.9 10*3/μL	0.9
Monocytes Absolute	0.3 to 0.8 10*3/μL	0.9
Glucose	74 to 100 mg/dL	108
Total Protein	6.3 to 8.2 g/dL	8.4
Total Bilirubin	0.2 to 1.3 mg/dL	1.5

At the current presentation, the patient described his pain as constant and stabbing in nature. He rated the pain 8/10 on a severity scale and exclaimed that the pain was aggravated by movement. He has reported multiple episodes of nausea, vomiting, and diarrhea since the onset. He denied having any fever, chest pain, shortness of breath, constipation, bloody stools, changes in diet, or a history of chronic gastrointestinal disease. The patient’s social history included cigarette smoking, recreational marijuana use, and no alcohol consumption.

The patient’s constitutional physical exam revealed a well-developed, well-groomed, and normal-weight adult male. His abdomen was flat and soft, with no masses on palpation, and he exhibited normal bowel sounds via auscultation. He also demonstrated palpable epigastric and periumbilical rebound tenderness with guarding. On further review of the systems, the patient was negative on all other findings outside his gastrointestinal signs and symptoms. Lipase was within normal limits. Abnormal lab findings are shown in Table 2.

**Table 2:** Abnormal lab results from February 22, 2023 WBC: White blood cell count

Component	Reference Range & Units	02/22/23
WBC	4.00 to 11.00 10*3/μL	12.24
Hematocrit	37.0% to 47.0%	48.3
Neutrophils Relative	41.0% to 79.0%	88.1
Lymphocytes Relative	15.0% to 48.0%	6.7
Neutrophils Absolute	2.2 to 4.8 10*3/μL	10.8
Glucose	74 to 100 mg/dL	131
Creatinine	0.55 to 1.02 mg/dL	1.05
Albumin	3.4 to 5.0 g/dL	5.4
Anion Gap	45061.00	16.0

**Figure 2:**
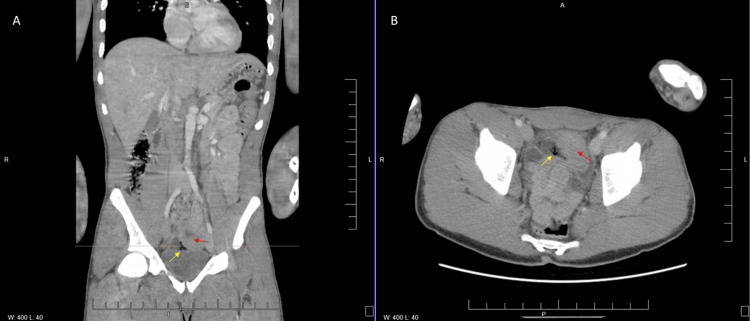
CT abdomen and pelvis with contrast from February 22, 2023, demonstrating air bubbles and bowel thickening A) CT abdomen and pelvis coronal view. The dotted orange line indicates the corresponding level in Panel B. Yellow arrows indicate a free-air bubble. Red arrows indicate bowel thickening. B) CT abdomen and pelvis axial view.

The patient was treated in the emergency department for pain, along with antiemetics, antacids, and x1 bolus of normal saline IV hydration. A CT of the abdomen pelvis with IV contrast revealed a mass of thickened bowel, mesenteric fat, and vascular pedicle within the midline pelvis (Figure 2). The mass of thickened bowel extended anteriorly and inferiorly, reaching just above the bladder dome in the sagittal plane. Within this vicinity and lower within the pelvis, imaging revealed areas of free fluid and small bubbles of free air at multiple locations (Figure 2). Suspicion of possible intestinal intussusception or internal hernia, as well as bowel ischemia, required urgent surgical consultation for a possible exploratory laparotomy.

Surgical intervention

On the day of arrival at the emergency department, an exploratory (diagnostic) laparoscopy (ex-lap) was indicated based on the patient's clinical presentation and radiographic findings of free air and free fluid within the pelvis. The patient was kept on nothing per mouth (NPO) and given intravenous (IV) normal saline upon admission. The patient was taken to the surgical suite in the afternoon and was administered general anesthesia with the placement of an endotracheal tube. ChloraPrep was used on the abdomen for sterilization, with sterile draping placed afterward. An official time-out was called with all in agreement, and the patient received 2g of prophylactic cefazolin immediately before the incision. The general surgeon placed the veress needle in the left upper abdominal quadrant and insufflated the abdominal cavity with CO2 at 1-2 L/min to reach an intrabdominal pressure of 12 mmHg. Three laparoscopic trocars were placed, and the bowel was inspected.

**Figure 3:**
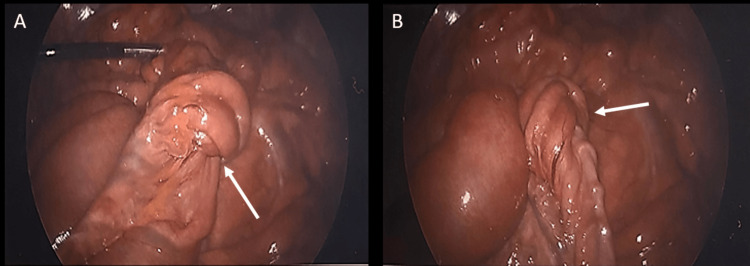
Intussusception in the distal ileum A) Initial laparoscopic view of ileo-ileal intussusception (white arrow) in the distal ileum. B) Additional view of intussusception (white arrow) in the distal ileum clearly showing the bowel telescoping.

**Figure 4:**
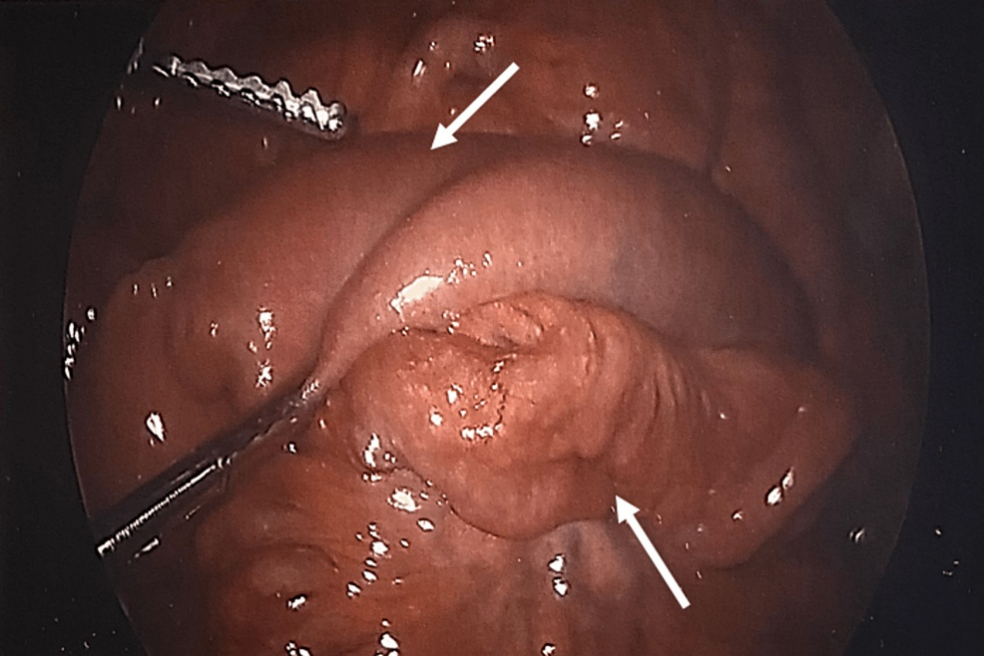
Reduced distal ileum intussusception, demonstrating dilated distal bowel Laparoscopic view. A white arrow in the upper region demonstrates dilated distal bowel. The white arrow in the bottom region shows the now-reduced ileo-ileal intussusception.

An incidental ileo-ileal intussusception was discovered in the distal ileum with no leading tumor, polyp, or identifiable mass (Figure 3). The remainder of the small and large bowels were carefully inspected for any other irregularities, such as masses or perforations, but none were found. A manual reduction of the intussusception was successfully accomplished (Figure 4). Visible peristalsis of the dilated bowel with no areas of necrosis or spillage was seen, so the patient was sent to recovery with no further surgical intervention. In his postoperative management, the patient was kept on a clear liquid diet and IV saline for two days until he was discharged. On postoperative day two (POD2), the patient was sent for an abdominal magnetic resonance elastography (MRE). The MRE analysis showed no evidence of bowel obstruction or acute inflammatory changes. There were no ascites or lymphadenopathies. Despite an atherosclerotic aorta, imaging analysis deemed the aorta to be of normal caliber, along with a patent IVC and a normal MRCP. Overall, the abdominal MRE was evaluated to be normal, specifically with no evidence of an abdominal mass and a successful reduction of the intussusception.

## Discussion

Adult intussusception is a rare clinical entity, accounting for only 1%-5% of all cases of intestinal obstruction [5,11]. Of these, the vast majority (90%) of cases can be attributed to an identifiable neoplasm, and the remainder (10%) are idiopathic [6,9]. While the exact mechanism is still unknown, it is believed that a pathological lead point or lesion within the bowel wall alters normal peristaltic activity [11], leading to the invagination of a proximal segment of the bowel into a distal segment, resulting in obstruction [1-6]. Other predisposing factors for small bowel intussusception in adults include anorexia nervosa and malabsorption syndromes due to the increased flaccidity of the bowel wall, which facilitates invagination, and supratherapeutic anticoagulation therapy, which may cause submucosal hemorrhages that can lead to intussusception [6]. Adult intussusception can also be caused iatrogenically due to the presence of intestinal tubes, jejunostomy feeding tubes, or after gastric surgery [5].

One method for classifying intussusceptions is based on their location in the gastrointestinal tract: (1) entero-enteric, confined to the small bowel; (2) colo-colic, confined to the large bowel; (3) entero-colic, the prolapse of the terminal ileum into the ascending colon; or (4) ileo-cecal, where the ielo-cecal valve is the lead point [5]. Entero-colic intussusception is the most common type [6].

Intussusceptions can also be classified according to etiology (benign, malignant, or idiopathic) [5,9,11]. A majority of identifiable neoplasms causing intussusception are benign (80%), such as adhesions, lymphoid hyperplasia, lipomas, Meckel’s diverticulum, Peutz-Jegher adenoma, gastrointestinal stromal tumors, hemangiomas, trauma, or leiomyomas [9]. Malignant lesions, on the other hand, account for 20%-30% of intussusception cases in the small bowel but represent up to 66% of cases in the large bowel [5,7]. Rarely, there have also been reported cases of adult intussusception being caused by non-Hodgkin’s lymphoma and Kaposi’s sarcoma [5,9].

The clinical presentation of adult intussusception varies considerably. In pediatric patients, the classic presentation of intussusception consists of a triad of cramping abdominal pain, a palpable tender mass, and bloody diarrhea [5]. In adults, however, most patients present with nonspecific symptoms that are either subacute or chronic and are more consistent with a partial obstruction. The most common presentation includes nausea, vomiting, abdominal pain, and bleeding per rectum [11]. Abdominal masses are palpable in only 24%-42% of adult patients, and identification of a shifting mass or one that is palpable only when symptoms are present is suggestive of intussusception [7,9]. The presentation of intussusception in adults is so vague and nonspecific that a clinical diagnosis beyond bowel obstruction is rarely made before surgery [5].

Several imaging techniques have been described as useful for diagnosing adult intussusception [5-7,9,11]. Plain abdominal films are typically the first diagnostic tool, which may demonstrate signs of intestinal obstruction or provide information regarding the site of obstruction, but further imaging is typically required. Ultrasonography is the principal radiological procedure used in pediatric patients, but it can also be utilized in diagnosing intussusception in adults. The classical imaging features include the 'target' or 'doughnut' sign on the transverse view and the 'pseudo-kidney' or 'hay-fork' sign on the longitudinal view. A significant disadvantage to using this technique in adults is the masking of the intussusception by gas-filled bowel loops, which heavily depends on the operator's ability. In recent years, abdominal CT has been reported as the most useful diagnostic tool for intussusception and has become the preferred imaging technique, after plain abdominal X-rays, in evaluating patients with nonspecific abdominal complaints. An intussusception can appear as a 'target sign' on a perpendicular view or as a sausage-shaped mass on the longitudinal axis, along with mesenteric fat and vessels. However, these classic findings may not be seen on a CT scan. Some of the vaguer characteristics, such as bowel thickening, air within the bowel, mesenteric fat, and vessels, can be seen in other pathologies, such as intramural hematomas, internal hernias, volvulus, or bowel ischemia [6,12]. There has also been a case reported in which the classic target sign found on CT imaging was thought to be an intussusception, but exploratory laparotomy demonstrated a large phytobezoar that was noted to be obstructing the ileocecal valve instead [13]. CT scans are also limited by less accessibility, static and single-plane exploration, radiation, and oral and IV contrast, which could further delay the study and, thus, the proper diagnosis [6]. Consideration should be made that, with advancing CT scans, nonpathological transient short-segment intussusceptions are now commonly seen. An additional benefit of a CT scan is that it can also disclose the intussusception's location, the nature of the mass, and its relationship to surrounding tissue. It may aid in staging the patient with a suspected malignant pathological lead point [4].

Intussusception is almost always treated surgically in adults due to the risk of intestinal ischemia and possible malignancy of the lead point [11]. However, most cases are only established once the patient is in the operating room due to the patient’s presentation with nonspecific symptoms and vague imaging findings. Once an intussusception is diagnosed laparoscopically, the extent of bowel resection or mechanical manipulation of the intussuscepted bowel during reduction remains controversial [5]. Some authors advocate for reducing the intussusception before resection to try and limit the extent of bowel that will need to be resected. This especially holds true with idiopathic intussusception, where reduction without resection is the preferred method of treatment. However, this should not be attempted if there are signs of inflammation or ischemia of the bowel wall. In patients with a pathological lead point, reducing the bowel could expose the patient to the risk of seeding tumor cells, perforation, and an increased risk of anastomotic complications. However, no clear evidence exists on this issue [14].

## Conclusions

Intussusception is an uncommon occurrence in adults and typically presents with nonspecific symptoms, making preoperative diagnosis difficult. Abdominal CT is considered the most sensitive diagnostic imaging tool for adult intussusception and can help distinguish between the presence or absence of a pathological lead point. Because adult intussusception cases are frequently associated with malignant lesions, surgical resection is often necessary for treatment. This case highlights the rarer case of idiopathic adult ileo-ileal intussusception without a lead point. Treatment still required surgical intervention but was sufficiently treated with manual reduction without the need for resection. Further research must be done to identify better diagnostic tools for idiopathic intussusception in adults.
